# One-year survival after critical care as a decision basis for advance care directives in general medicine: Real word data analysis of 149,144 patients

**DOI:** 10.1371/journal.pone.0326031

**Published:** 2025-06-27

**Authors:** Constantin Unger, Felix Werner, Bettina Engel, Thomas Kühlein, Christoph Schulz, Christian Kümpel, Johannes Gorkotte, Susann Hueber

**Affiliations:** 1 Allgemeinmedizinisches Institut, Friedrich-Alexander-Universität Erlangen-Nürnberg, Erlangen, Germany; 2 GWQ Service Plus AG, Düsseldorf, Germany; Azienda Ospedaliero Universitaria Careggi, ITALY

## Abstract

Providing counsel on advance care directives is challenging for general practitioners. Counselling is done on unknown future circumstances of possible critical illness and critical care in intensive care units. Following the principles of evidence-based medicine, the physician’s task is to communicate evidence and elucidate the patient’s position on it. However, suitable evidence of chances of survival in case of critical illness is lacking. Aim of this study was to generate long-term survival rates of patients receiving critical care as evidence for general practitioners who provide counselling for patients on advance care directives. We conducted a retrospective cohort study analysing one-year survival rates of critical care using German health insurance claims data from an anonymised nationwide health claims data pool of over five million German patients. All patients over 18 years of age receiving critical care for the first time were included.Main outcome of our study were one-year survival probabilities depending on age and on acute life prolonging procedures. Procedures analysed were non-invasive and invasive mechanical ventilation (nMV, iMV), renal replacement therapy (RRT), their combinations (nMV + RRT, iMV + RRT), and cardiopulmonary resuscitation (CPR). A total of 149,144 datasets was analysed. One-year survival probability of all patients was 77.5%. Survival rates ranged from 94.5% in patients under 50 without any further acute life prolonging procedures to 16.4% in those older than 80 who received iMV + RRT. The application of at least one procedure was associated with an increased risk of death (HR 3.06, 95% CI 2.99 to 3.12) as was CPR (HR 4.22, 95% CI 4.07 to 4.37). Differences between pre- and COVID periods were modest. To enable patient’s decision-making in creating advance care directives, our results provide easily applicable external evidence for general practitioners counselling on advance care directives by providing probabilities of survival in critical care.

## Background

In his book “Being Mortal” the surgeon and writer Atul Gawande wrote: “The waning days of our lives are given over to treatments that addle our brains and sap our bodies for a sliver’s chance of benefit. They are spent in (…) intensive care units (…) where regimented, anonymous routines cut us from all the things that matter to us in life” [1]. This statement eloquently sums up one dilemma of modern medical care: While intensive care units (ICU) can be life-saving, almost one out of five patients dies despite all treatment, still in hospital [2]. Considering these possibly definite consequences for patients, medical decision-making in this context is especially challenging. According to the principles of evidence-based medicine patients’ preferences, the best available external evidence and individual clinical expertise of physicians should be integrated in a shared decision-making process [3]. However, eliciting patients’ preferences is frequently impossible in case of illness requiring critical care. Widely applied approaches seek to explore patients’ preferences in advance and document their decisions in so-called “advance care directives” [4]. While this concept might be beneficial for reducing health care costs [5] and these documents seem to introduce patients’ preferences into medical decision-making to some extent [4,6,7], there is a challenge for counselling physicians. Usually, they are not the same physicians who treat those patients in ICUs. The counselling on advance care directives is frequently done in an ambulatory setting by general practitioners (GPs) [8].

GPs are frequently uncertain about how to structure counselling patients and have little time to do so anyway [9,10]. Evidence for the efficacy of structured communication tools is scare [11]. Patients on the other hand expect their GPs to initiate the corresponding discussion [12]. During counselling on advance care directives, GPs need to convey possible benefits and harms of future critical care which might never become relevant to patients who are healthy enough to come to the physicians’ office and are influenced by an overly optimistic depiction of critical care procedures in fictious television shows [13]. To communicate those benefits and harms, chance of survival and giving up the things that matter in life [1], GPs need to estimate probability of survival associated with those measures realistically themselves, of course. Yet, primary care physicians such as GPs tend to overestimate survival probabilities in critical care [14,15].

Given possible limited clinical expertise of GPs in critical care which could be helpful during counselling [16] and patients being normally unfamiliar with critical care, applicable external evidence [3] can bridge the gap between the ambulatory setting and critical care in ICU and enable informed decision-making for advance care directives. While optimal external evidence for counselling must address patients’ individual therapy goals in the case of critical illness, long-term survival probabilities of patients in ICU serve as a fundamental starting point, as survival is a necessary condition for achieving any therapy goal.

Therefore, the aim of this study was to generate long-term survival rates of patients receiving critical care as an external evidence base for general practitioners who provide counselling for patients on advance care directives considering their age and the most common acute life-prolonging procedures in critical care.

## Methods

### Study design

To elucidate one-year survival rates of patients after ICU admission we conducted a survival analysis after intensive care using German claims data. For the analysis, an anonymised data pool was provided by GWQ Service Plus AG, a joint venture of medium-sized statutory health insurance funds. The data pool contains claims data of over five million German patients who are on public health insurance beginning in 2010 and is constantly kept up to date. The anonymised data pool is not publicly accessible. Due to the anonymisation of data, identifying individuals was impossible during the analysis. An ethical approval was not required, as German law allows for analysing anonymous routinely-collected data for research purposes without patients‘ consent. A corresponding statement was obtained (approval number: 24–310-ANF). The final data access was performed on October 19^th^, 2023. Information regarding ICU stays and life-prolonging procedures were obtained from the German Procedure Classification (Operationen- und Prozedurenschlüssel [OPS]) [17] and the out-patient accounting cypher (Einheitlicher Bewertungsmaßstab [OAC]) [18]. Obtained OPS codes depict the most commonly used acute life-prolonging procedures in ICU which are also considered in advance care directive forms [19], namely non-invasive mechanical ventilation (nMV), invasive mechanical ventilation (iMV) or renal replacement therapy (RRT) and their combinations (nMV + RRT, iMV + RRT). Additionally, we gathered OPS and OAC indicating the performance of cardiopulmonary resuscitation (CPR). The complete list of obtained OPS and OAC codes and their definitions and meanings are shown in Table 1.

**Table 1:** Obtained procedures German procedure classifications (OPS) and out-patient accounting cyphers (OAC) and corresponding attribution to acute life prolonging procedure; intensive care unit (ICU), non-invasive mechanical ventilation (nMV), invasive mechanical ventilation (iMV), renal replacement therapy (RRT), cardiopulmonary resuscitation (CPR).

ICU	OPS	8-980	Intensive care treatment
	OPS	8-98f	Complex intensive care treatment
**nMV**	OPS	8-713.0	Ventilatory support by high flow nasal cannula
	OPS	8-706	Ventilation by face mask
**iMV**	OPS	8-701	Normal endotracheal intubation
	OPS	8-704	Intubation with double-lumen endotracheal tube
	OPS	5-311	Temporary tracheostomy
	OPS	5-312	Permanent tracheostomy
**RRT**	OPS	8-853	Hemofiltration
	OPS	8-854	Hemodialysis
	OPS	8-855	Hemodiafiltration
**CPR**	OPS	8-77	Resuscitation procedures
	EBM	01220	Out-of-hospital resuscitation

Derived from our study rationale, we did not obtain information to compare survival probabilities of treated and untreated patients. Furthermore, we did not consider diagnoses leading to ICU admission because in the moment of counselling it is usually unknown if and how critical illness occurs. While already known chronic conditions could be relevant in the moment of counselling, we did not consider this information in our analysis to avoid bias as underlying data – ICD-10 codes of chronic conditions – were imprecise in that regard [20,21]. Beyond simply indicating whether a procedure was performed or not like OPS, ICD-10 allows for a more distinct coding of diseases which may often be done heterogeneously, party driven by reimbursement considerations, and frequently outdated. The study reporting is based the German Good Practice Secondary Data Analysis (GPS) guidelines and recommendations [22] as well as on the Strengthening the Reporting of Observational studies in Epidemiology (STROBE) reporting guideline and the Reporting of studies Conducted using Observational routinely-collected health Data (RECORD) statement [23]. Patients or the public were not involved in our research.

### Study population

All patients over 18 years of age with an ICU stay were eligible. Inclusion period started on January 1^st^ of 2012, if patients had not been admitted to the ICU or had any of selected procedures (Table 1) in at least the two assessable years before. The study population selection is shown in S1 Fig. This exclusion timeframe of at least two years was chosen because possible impacts of prior critical illness more than two years ago were considered having only small influence on survival probability [2] without being overly restrictive and reducing sample size. A proportion of German ICU patients is admitted electively for surveillance and only for a short time period especially after surgery or other interventions. To be selective of acute critically-ill patients we defined that the ICU stay had to be over 24 hours, also accordingly to OPS code for the ICU. All OPS and OAC codes of interest during this ICU stay were obtained. As nMV is frequently used before or after iMV, patients with OPS code for both were defined as patients receiving iMV. Inclusion period ended on December 31^st^, 2021. In case of loss of follow-up datasets were censored.

### Statistical analysis

Patient characteristics included age, sex, and OPS codes as well as OAC codes for acute life prolonging procedures, also describing frequency of any RRT and CPR. Cohorts were formed according to age groups (18−50 years, 51−65 years, 66−80 years, > 80 years) and treatment with acute life prolonging procedures (none, nMV, iMV, RRT, and combinations nMV + RRT, iMV + RRT). For all groups mortality rates were computed for 90 day-intervals as well as on day 365. Kaplan-Meier-Curves were used to describe survival probability for all treatment cohorts and the age groups according to applied acute life prolonging procedure. Cox regression models were applied to estimate hazard ratios of any life prolonging procedures versus none as well as association with CPR either before or during hospital stay. To determine if there were any differences between the pre- and the COVID-19 period, data of patients admitted to ICU pre- or during COVID time were compared with each other (before and after March 1^st^ 2020). Frequencies are described in total numbers and corresponding percentages. When appropriate median values and interquartile ranges were used. Differences between the groups were compared with standardized mean difference [24]. Statistical analysis was performed using R Statistical software version 4.1.2.

## Results

### Study population

We included data of 149,144 patients in our analysis. The overall rate of male patients was 60.9%, median age was 64 years (IQR 53–74). A total of 1,928 (1.3%) cases was lost to follow-up. The age group from 18 to 50 years contained 31,179 patients (20.9%), the age group from 51 to 65 years 47,557 patients (31.9%), the age group from 66 to 80 years 54,324 patients (36.4%) and the age group over 80 years 16,084 patients (10.8%). The relative number of male patients was highest in patients from 51 to 65 years of age (65.5%) and decreased over the age groups.

Over all age groups, a total of 109,068 (73.1%) patients were in ICU without any further acute life prolonging procedure. nMV and iMV occurred in 8.3% and 13.6% of all patients, respectively. A total of 1.8% of all cases was treated with only RRT, whereas 0.6% of the study population was also treated with nMV (nMV + RRT) and 2.6% with iMV (iMV + RRT). Aggregated, 5.0% of all patients received RRT alone or in combination with respiratory support. An amount of 4.2% of the cases went through CPR.

Separated into age groups, the number of patients in the ICU without any procedure was highest in the group under 50 years of age (77.6%). Application of nMV doubled from youngest to oldest age groups (5.3% to 10.9%), while iMV was used most frequently in the age group from 51–65 years (14.2%) and least frequently in patients older than 80 (11.3%). RRT and combinations with nMV and iMV were used slightly more often in the groups from 51 to 65 and 66–80 years than in youngest and oldest patients. CPR was performed in 3% of patients aged 18–50, in 4.2% in aged 51–65 and in 4.8% of the remaining two age groups. Sociodemographic characteristics and amounts of procedures for all patients and age groups are shown in Table 2.

**Table 2:** Study population; intensive care unit (ICU), non-invasive mechanical ventilation (nMV), invasive mechanical ventilation (iMV), renal replacement therapy (RRT), cardiopulmonary resuscitation (CPR).

	Overall	18-50 years	51-65 years	66-80 years	>80 years
**n** (%)	149,144 (100)	31,179 (20.9)	47,557 (31.9)	54,324 (36.4)	16,084 (10.8)
**Median age** (IQR)	64 (53-74)	43 (34-47)	58 (55-62)	73 (69-77)	84 (82-86)
**Male** (%)	90,772 (60.9)	18,769 (60.2)	31,161 (65.5)	32,952 (60.7)	7,890 (49.1)
**Only ICU** (%)	109,068 (73.1)	24,209 (77.6)	34,618 (72.8)	38,410 (70.7)	11,831 (73.6)
**nMV** (%)	12,452 (8.3)	1,646 (5.3)	3,815 (8.0)	5,237 (9.6)	1,754 (10.9)
**iMV** (%)	20,239 (13.6)	4,222 (13.5)	6,733 (14.2)	7,467 (13.7)	1,817 (11.3)
**RRT** (%)	2,726 (1.8)	378 (1.2)	818 (1.7)	1,201 (2.2)	329 (2.0)
**nMV + RRT** (%)	837 (0.6)	84 (0.3)	231 (0.5)	415 (0.8)	107 (0.7)
**iMV + RRT** (%)	3,822 (2.6)	640 (2.1)	1,342 (2.8)	1,594 (2.9)	246 (1.5)
**all RRT combined** (%)	7,385 (5.0)	1,102 (3.5)	2,391 (5.0)	3,210 (5.9)	682 (4.2)
**CPR** (%)	6,302 (4.2)	936 (3.0)	1,975 (4.2)	2,619 (4.8)	772 (4.8)

### Survival probabilities

**Fig 1:**
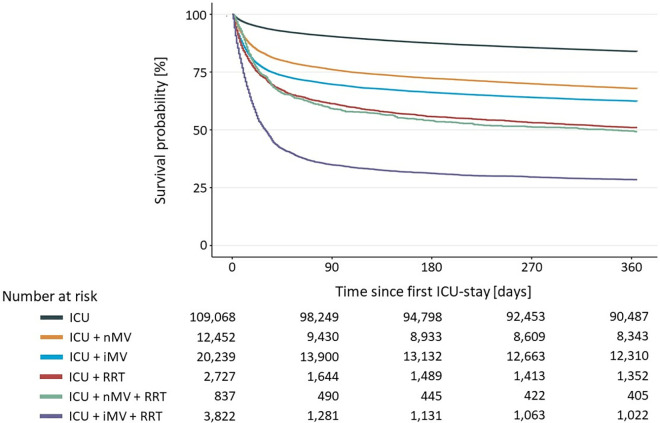
Kaplan-Meier-Curve for all patients from ICU admission to 360 days after.

Overall, one-year-survival probability was 77.5%. There were no statistically significant differences in mortality of men and women (HR 1.00, 95%-CI 0,98–1,03, *p = 0.79).* Survival probability decreased with age from 91.1% (18–50 years of age) to 83.3% (51–65 years) to 71.8% (66–80 years) to 53.4% (older than 80). Survival rates declined from subgroups with no procedure during ICU-stay to those with one procedure to those with a combination of procedures (nMV/iMV + RRT). Survival probabilities of subgroups with one procedure were highest associated in those with nMV and lowest in those with RRT. There was sharp decline of the survival probability in the subgroup with iMV + RRT compared to any other subgroup. Survival probabilities for ICU and any acute life prolonging procedures and their combinations are shown in Fig 1.

The survival rate for patients of all age groups who receive only intensive care admission but none of the predefined procedures is 90.1% at 90 days and falls to 83.0% at 360 days. By contrast, the survival rate for patients who receive both invasive mechanical ventilation and renal replacement therapy is 33.5% at 90 days and 26.7% at 360 days.

**Fig 2:**
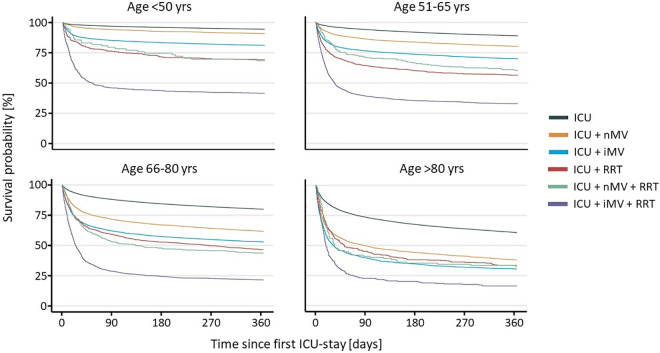
Kaplan-Meier-Curve for all age groups from ICU admission to 360 days after.

Over all age groups these trends were consistent with the exception of a lower survival probability of RRT only (56.4%) compared to RRT + nMV (60.2%) in patients aged 51–65. Considering all subgroups, one-year survival probability ranged from 94.5% in patients aged 18–50 in the ICU without any procedure to 16.3% of patients over 80 with iMV + RRT. Survival probabilities of all subgroups are shown in detail in Table 3 and Fig 2.

**Table 3:** 1-year-survival probabilities for age groups overall and subgroups of acute life prolonging procedures; intensive care unit (ICU), non-invasive mechanical ventilation (nMV), invasive mechanical ventilation (iMV), renal replacement therapy (RRT), cardiopulmonary resuscitation (CPR).

	Overall	ICU only	nMV	iMV	RRT	nMV + RRT	iMV + RRT
**18-50 years** (n included)	31,179	24,209	1,646	4,222	378	84	640
*1-year survival probability (%)*	91.1	94.5	90.9	81.1	69.3	68.4	41.5
**51-65 years** (n included)	47,557	34,618	3,815	6,733	818	231	1,342
*1-year survival probability (%)*	83.3	88.9	80.2	70.0	56.4	60.2	32.9
**66-80 years** (n included)	54,324	38,410	5,237	7,467	1,201	415	1,594
*1-year survival probability (%)*	71.8	80.0	61.7	52.8	46.6	43.7	21.5
**> 80 years** (n included)	16,084	11,831	1,754	1,817	329	107	246
*1-year survival probability (%)*	53.4	60.7	38.0	30.4	33.5	32.5	16.3

As the age group increases, the probability of survival in the first 360 days after admission to an intensive care unit markedly declines. Furthermore, the impact of intensive care procedures is more pronounced in older age groups.

The application of at least one procedure was associated with an increased risk of death (HR 3.06, 95%-CI 2.99 to 3.12, *p < 0.001*) as was necessity of CPR (HR 4.22, 95%-CI 4.07 to 4.37, *p < 0.001*).

### Pre-COVID-19- and COVID-19-periods

Overall, one-year survival probability was 77.7% for the pre-COVID-19- and 76.6% for the COVID-19-period. The cohort after the beginning of the COVID-19-pandemic had a similar mean age as the cohort before the pandemic (median age 65, IQR 52–74, *vs.* 64, IQR 55–75) and contained more men (61.3% *vs.* 60.7%). Relative numbers of the age groups between 18–50 (21.7% *vs.* 18.2%) and 66–80 (36.8% *vs.* 35.1%) years decreased after the beginning of the COVID-19-pandemic, while numbers of patients aged 51–65 (31.3% *vs.* 34.1%) and over 80 (10.3% *vs.* 12.6%) increased. Appliance of acute life prolonging procedures was relatively constant. Most distinct differences were in a decrease of patients with only ICU (74.2% *vs.* 69.4%) and an increase of nMV (7.1% *vs.* 12.8%). Standardized mean difference was lower than 0.2 over all subgroups (S1 Table).

## Discussion

For all patients included, one-year survival probability was 77.5%. Survival rates decreased over the age groups from 91.1% in patients aged 18–50 to 53.4% in those older than 80. Probability of survival also decreased with the application of acute life prolonging procedures compared to no procedures necessary and was highest in patients with iMV + RRT. Resulting probabilities ranged from 94.5% in patients aged 18–50 without procedures to 16.4% in those older than 80 receiving iMV + RRT. Association with CPR also decreased probability of survival. Although the COVID-19 pandemic seemed to have an impact on health care, differences in one-year-survival after an ICU stay were small.

While survival probabilities differed widely and decreased steeply in those older than 80, differences between application rates of acute life prolonging procedures over the age groups were relatively small. Slightly lower frequencies of acute life prolonging procedures were found in patients aged 18–50 and in those older than 80. These lower frequencies might be explained either by reduced necessity of acute life prolonging procedures or by therapy limitation. It is plausible that younger patients were more likely to survive without any further procedures, whereas therapy of older patients was more frequently limited due to unfavourable prognosis. In line with that, the highest relative amount of nMV and the lowest of iMV in patients over 80 might reflect a higher probability for the decision not to escalate critical care beyond nMV. Frequency of CPR on the other hand, was not lower in patients over 80. This could indicate a lower probability of therapy limitation in highly acute situations.

Alongside the steep decrease of survival in patients older than 80, there was another steep decline of survival in patients who were treated with iMV + RRT. Furthermore, as shown in Figs 1 and 2, survival probabilities declined especially in the first months after index ICU admission. These changes of survival probability in dependence of clinical circumstances and over time could be a fitting starting point in counselling to discuss the patient’s therapy goals different from mere survival. Realistic estimations of survival probability as provided can be used to support patients to better decide according to their personal values during creating advance care directives. When discussing survival probabilities associated with ICU stays and acute life prolonging procedures it is paramount to clarify that lower probabilities are not caused by the respective procedures. These probabilities are the chance of benefiting from critical care while accepting its harms. Patients who decline critical care are highly likely to die in case of critical illness.

One-year survival probabilities reported in previous studies with similar data sources and design vary from 67.8% in Taiwan [25] to about 70% in France [2] and approximately 75% in Canada [26]. Also, median age of the study population is in line with those comparable observational studies [2,25,26]. While age distribution over the study population is overall similar as described from France by Atramont et al. [2], we found a lower amount of patients over 80 years and a higher amount of patients between 51 and 80 years. In contrast to our findings, single-center studies described higher as well as lower 1-year mortality rates in patients over 80 [27,28]. Frequency of acute life prolonging procedures differs widely across studies [2,25–27]. In comparison, our findings showed lower rates of acute life prolonging procedures, especially of MV. About 60% of included patients were male, also comparable to previous studies [2,25,26]. This disparity in gender distribution disappeared in patients above 80 years, as described before [27]. The highest overall one-year survival rates and the tendency to a younger study population with less application of life prolonging procedures in our study fits our selection of patients without prior critical illness. Additionally, differences in study design as well as in respective health care systems might be of influence. However, higher age and appliance of life prolonging procedures were relatively consistent associated with lower survival probabilities [2,25–27]. Therefore, we assume the differences in age and procedure subgroups to be smaller than in overall survival. Results of a recent German study which analysed in-hospital mortality of mechanically ventilated patients using also claims data could indicate this tendency while being limited in comparability [29]. To specify this and provide external evidence as accurate as possible for counselling about advance care directives, further research in other countries than Germany is necessary.

Our study had several limitations and strengths. We were not able to confidently differentiate between elective and emergency ICU admissions. Although we tried to accommodate for this by defining ICU stay as at least 24 hours, high amounts of patients without any life prolonging procedure suggest relevant numbers of remaining patients admitted electively. Therefore, we also did not consider patients who died within the first 24 hours after ICU admission. In result, especially overall survival probabilities might be overestimated in our study. Our focus on patients without critical illness prior to the index ICU admission might add to that and reduces generalizability for patients with history of critical illness. Because of our selection process and a high number of subgroups analysed, some survival analyses were performed on relatively low numbers.

We accessed data if a case was associated with CPR though we did not obtain any further information regarding CPR like time without thorax compressions or if cardiac arrest was observed. Therefore, the provided evidence is limited to the fact that performed CPR is associated with further decreased survival probability.

If patients want to consider known chronic conditions when deciding about their advance care directives, our results do not allow counselling GPs to specify possible influence on survival probability. However, in that case GPs can integrate provided external evidence with their clinical expertise and knowledge of the patient’s history to consider details in counselling. To exactly determine the influence of chronic conditions on survival probability, further research is necessary. Additionally, our results are only partly applicable to patients who had already been admitted to the ICU prior to counselling. Also, to elucidate the impact of prior admissions to ICU on survival probabilities in case of recurring critical illness, further research is required.

Our study focused solely on survival and does not provide any empirical measurements regarding other therapy goals. Yet, further therapy goals like physical independence or social participation should be explored during counselling. Although our study does not provide corresponding probabilities for reaching other therapy goals, we explicitly encourage physicians to discuss those therapy goals. Finally, we selected only patients ensured by cooperating health insurances. Some information relevant for counselling like possible influence of existing advance care directives on prognosis are not available in health claims data.

A major strength of our study is the high number of patients included from a nationwide dataset and only few cases of loss of follow-up. Our focus on most commonly used life prolonging procedures in critical care and an end-point comprehensible for physicians and patients alike easily allow the application of our results in counselling in an ambulatory setting.

## Conclusion

Our study provides one-year survival probabilities for patients in the ICU for more than 24 hours depending on their age and the application of treatment with acute life prolonging procedures. Following the principles of evidence-based medicine, our results are an easily applicable evidence basis for general practitioners counselling on advance care directives and fundamental to decisions about advance care directives considering chances of survival in critical illness. As declining critical care and acute life prolonging procedures in most cases will lead to dying, of course, the data provided can be used for graduated decisions by weighing possible benefits and harms for the stay in the ICU itself and every acute life prolonging procedure. For GPs providing counsel in creating advance care directives, survival probabilities are an intuitive starting point of discussing individual therapy goals with patients, which can be quite different from mere survival. Further research is necessary to evaluate how to incorporate our results in counselling on advance care directives optimally.

## Supporting information

S1 Fig Flowchart of the study population selection. (DOCX)

S1 Table Comparison of the study population during pre-COVID and COVID-period. (DOCX)

S1 Checklist RECORD checklist. (PDF)

## Abbreviations

CPR: Cardiopulmonary resuscitation
OAC: Out-patient accounting cypher (Einheitlicher Bewertungsmaßstab, EBM)
ICU: Intensive Care Unit
iMV: Invasive mechanical ventilation
nMV: Non-invasive mechanical ventilation
OPS: German Procedure Classification (Operationen- und Prozedurenschlüssel)
RRT: Renal replacement therapy.
