# Correction: One-year survival after critical care as a decision basis for advance care directives in general medicine: Real word data analysis of 149,144 patients

**DOI:** 10.1371/journal.pone.0335849

**Published:** 2025-10-30

**Authors:** Constantin Unger, Felix Werner, Bettina Engel, Thomas Kühlein, Christoph Schulz, Christian Kümpel, Johannes Gorkotte, Susann Hueber

The word “world” is misspelled in the article title. The correct title is: One-year survival after critical care as a decision basis for advance care directives in general medicine: Real world data analysis of 149,144 patients. The correct citation is: Unger C, Werner F, Engel B, Kühlein T, Schulz C, Kümpel C, et al. (2025) One-year survival after critical care as a decision basis for advance care directives in general medicine: Real world data analysis of 149,144 patients. PLoS One 20(6): e0326031. https://doi.org/10.1371/journal.pone.0326031
