# Supplementary figures and images for: One-year survival after critical care as a decision basis for advance care directives in general medicine: Real word data analysis of 149,144 patients
Source: PLoS One. 2025 Jun 27;20(6):e0326031. doi: 10.1371/journal.pone.0326031 (PMC12204473; doi:10.1371/journal.pone.0326031)

**S1 Fig. Flowchart of the study population selection**


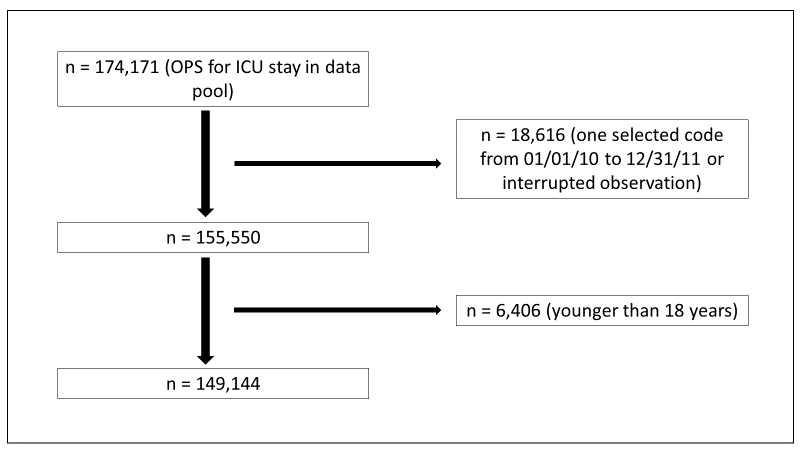

Supplement: S1 Fig — (DOCX) [file pone.0326031.s001.docx]
