# Supplementary material for: One-year survival after critical care as a decision basis for advance care directives in general medicine: Real word data analysis of 149,144 patients
Source: PLoS One. 2025 Jun 27;20(6):e0326031. doi: 10.1371/journal.pone.0326031 (PMC12204473; doi:10.1371/journal.pone.0326031)
Supplement: S1 Table — (DOCX) [file pone.0326031.s002.docx]

**S1 Table. Comparison of the study population during pre-COVID and COVID-period**

|  | **Pre-COVID period** | **COVID period** | **Standardized mean difference** |
| --- | --- | --- | --- |
| **n (%)** | 116,609 (78.2) | 32,535 (21.8) | - |
| **Age (SD), years** | 62.4 (15.3) | 63.0 (15.0) | 0.038 |
| **18-50 years (%)** | 25,259 (21.7) | 5,920 (18.2) | 0.087 |
| **51-65 years (%)** | 36,454 (31.3) | 11,103 (34.1) | 0.061 |
| **66-80 years (%)** | 42,903 (36.8) | 11,421 (35.1) | 0.035 |
| **>80 years (%)** | 11,993 (10.3) | 4,091 (12.6) | 0.072 |
| **Male (%)** | 70,826 (60.7) | 19,946 (61.3) | 0.012 |
| **ICU (%)** | 86,477 (74.2) | 22,591 (69.4) | 0.105 |
| **nMV (%)** | 8,284 (7.1) | 4,168 (12.8) | 0.191 |
| **iMV (%)** | 16,124 (13.8) | 4,115 (12.6) | 0.035 |
| **RRT (%)** | 2,110 (1.8) | 616 (1.9) | 0.006 |
| **nMV + RRT (%)** | 554 (0.5) | 283 (0.9) | 0.048 |
| **iMV + RRT (%)** | 3,060 (2.6) | 762 (2.3) | 0.018 |
| **CPR (%)** | 5,039 (4.3) | 1,263 (3.9) | 0.022 |
